# Dietary Supplements’ Regulation: Are We Doing Enough to Protect the Children? A Narrative Review

**DOI:** 10.3390/children13010074

**Published:** 2026-01-02

**Authors:** Jelena Jovičić-Bata, Nataša Milošević, Neda Gavarić, Maja Grujičić, Bojana Arsenov, Milana Vuković, Nemanja Todorović, Mladena Lalić-Popović

**Affiliations:** 1 Department of Pharmacy, Faculty of Medicine, University of Novi Sad, Hajduk Veljkova 3, 21000 Novi Sad, Serbia; jelena.jovicic-bata@mf.uns.ac.rs (J.J.-B.); neda.gavaric@mf.uns.ac.rs (N.G.); 1218d17@mf.uns.ac.rs (B.A.); milana.vukovic@mf.uns.ac.rs (M.V.); nemanja.todorovic@mf.uns.ac.rs (N.T.); mladena.lalic-popovic@mf.uns.ac.rs (M.L.-P.); 2 Department of General Education Subjects, Faculty of Medicine, University of Novi Sad, Hajduk Veljkova 3, 21000 Novi Sad, Serbia; maja.grujicic@mf.uns.ac.rs

**Keywords:** children, supplements, regulation, health claims, European Union, Serbia

## Abstract

Background: As the overall prevalence of dietary supplements (DS) use in pediatric populations is high, these products should be strictly regulated. However, regulatory frameworks for DS in the European Union and Serbia share inconsistencies and ambiguities which may compromise pediatric DS safety. Objective: To identify and critically assess the regulatory and practical issues in defining, labeling, advertising, and use of pediatric dietary supplements in the EU and Serbia. Methods: This review focused on identifying and assessing inconsistencies, gaps, and other regulatory challenges, as well as marketing practices affecting consumer safety through the assessment of legal and policy frameworks of the European Union and Serbia, and peer-reviewed articles, pertaining to the definition, labeling, and advertising of pediatric dietary supplements, that were assessed for contextual evidence on related, evidence-based, and practice-based information. Results: The analysis identified five critical areas of concern within the current regulations for pediatric DS: (i) the absence of universal and clear product definition, (ii) the lack of uniform, age-appropriate composition standards, (iii) potential safety risks related to ambiguous composition standards, arbitrary age cut-offs, and lack of age-appropriate reference values specific to DS, (iv) misuse of labels and unfair advertising practices, and (v) practical aspects of pediatric DS use and the limited role of healthcare providers in it. Conclusions: Regulating pediatric DS is a complex task due to the diversity of the pediatric population. Regulatory systems must be ready to swiftly resolve all inconsistencies and adjust to new scientific developments and market changes in order to ensure our primary goal—children’s health and safety.

## 1. Introduction

Dietary supplements (DS, also known as food supplements or nutrition/al supplements) market has been steadily rising for years worldwide. A predicted growth of the global DS market from USD 179.9 billion in 2025 to USD 318.46 billion by 2033 makes the expected compound annual growth rate 7.4% from 2026 to 2033 [1].

The driving force behind the rising DS demand is the aging population and the ever-more popular self-care trend, coupled with belief that DS are efficient and safe. Additionally, DS are frequently suggestively, even aggressively, promoted via mass-media, especially via social media and influencers, and are readily available for purchase [2,3]. The coronavirus pandemic, with consequent movement restrictions and curfews, stimulated online purchases of many consumer goods, including DS. This shift from traditional commerce models to e-channels further distanced consumers from competent healthcare professionals.

Frequently reported reasons for DS use are general wellness and “disease treatment” [4], but, among children, the immunity-boosting products are currently some of the most sought-after from this category [5]. The demand for multivitamin-multimineral DS, as well as singled-out vitamins or minerals, is regularly high [6,7,8,9]. Also frequently used among children are probiotics, n-3 fatty acids, amino acids, and melatonin [10,11]. Herbal dietary supplements are often used among children and adolescents in cases of coughs and colds, diseases of the oral cavity and pharynx, gastrointestinal disorders, urogenital tract complaints, skin diseases and minor wounds, mental stress and mood disorders, and pain and inflammation [12].

The overall prevalence of DS use in pediatric populations is high, averaging around 30–50% [6,7,9,13,14], with up to 79% of small children reported to be DS users [9]. With so many children consuming DS, it is of utmost importance for these products to be well-regulated. However, this is currently not the case.

Regulatory frameworks concerning DS are not globally harmonized and seem to be more oriented towards the market than the consumers. Currently, Serbian national legislation on DS is being harmonized with EU regulations. Therefore, the issues raised in the following text pertain to both regions.

The aim of this review was to identify and critically assess the regulatory and practical issues in defining, labeling, advertising, and use of pediatric dietary supplements in EU and Serbia.

## 2. Materials and Methods

This study is a narrative review identifying, summarizing, and critically discussing the legal and policy frameworks of the European Union and the Republic of Serbia pertaining to the definition, labeling, and advertising of pediatric dietary supplements. The analysis focused on identifying and assessing inconsistencies, gaps, and other regulatory challenges, as well as marketing practices affecting consumer safety.

Data sources included EU Regulations and Directives [15] and related guidance documents, as well as Serbian laws and by-laws governing DS [16]. These documents were screened for relevant provisions and information on product definition, composition standards, labeling requirements, use of health claims, safety, and advertising rules. In addition, a search of the peer-reviewed literature was conducted using PubMed and Scopus on 7 November 2025. The search was limited to articles in English, but with no date restriction. Truncation was applied using the asterisk to capture variations in word endings, while spelling variants were addressed by explicitly including both British and American forms (e.g., pediatric and paediatric; labeling and labelling). The following search string was used in PubMed: (“dietary supplement*” OR “nutrition* supplement*”) AND (infant* OR child* OR pediatric* OR paediatric* OR adolescent*) AND (use OR safety OR risk* OR labeling OR labelling OR advertising). In Scopus, the search string was adapted to database-specific syntax as follows: TITLE-ABS-KEY (“dietary supplement*” OR “nutrition* supplement*”) AND TITLE-ABS-KEY (infant* OR child* OR pediatric* OR paediatric* OR adolescent*) AND TITLE-ABS-KEY (use OR safety OR risk* OR labeling OR labelling OR advertising). Adult studies were not excluded at this search stage because they were needed for contextual comparison with pediatric findings.

Initial searches retrieved close to around 900 publications via PubMed and approximately 3300 publications via Scopus. Firstly, publications’ titles and abstracts were screened for relevance to the review objectives. Secondly, full texts of the selected publications were assessed by two researchers. Relevance was determined based on the extent to which a source provided regulatory or practical evidence related to the review objective. Discrepancies were resolved through discussion.

A total of 67 publications were included in the narrative synthesis, 47 of which are peer-reviewed publications, 8 are regulatory documents from EU and Serbia, whereas the remaining 12 are guidance documents or other publications relevant to the discussion.

Given the narrative nature of the review, no formal inclusion or exclusion criteria were applied.

To accommodate for the heterogeneous nature of the selected sources, thematic content analysis was selected as a suitable method for organizing and interpreting qualitative information. A thematic content analysis was performed using an inductive approach, following the Braun and Clarke framework [17]. Two researchers independently coded the source materials and then discussed discrepancies to reach consensus. In cases in which the consensus could not be reached, the principal investigator reviewed the data and made the final decision. Initial codes were grouped into broader categories, which were classified under five main themes: (i) the absence of universal and clear product definition, (ii) the lack of uniform, age-appropriate composition standards, (iii) potential safety risks related to ambiguous composition standards, arbitrary age cut-offs, and lack of age-appropriate reference values specific to DS, (iv) misuse of labels and unfair advertising practices, and (v) practical aspects of pediatric DS use.

An overview of identified themes and subthemes is presented in Table S1 in the Supplementary Material. Table S2 illustrating examples of thematic coding derived from regulatory documents and the selected literature on pediatric dietary supplements is provided in Supplementary Material.

## 3. Results and Discussion

### 3.1. The Absence of Universal and Clear Pediatric Dietary Supplements Definition

There is no international consensus on the definition of DS [18] but the core concepts in most are that DS are products intended for oral use, designed to address nutrient gaps. At the moment, in the EU and Serbia, DS are defined as foods supposed to supplement the diet or, more specifically, “concentrated sources of nutrients (i.e., vitamins, minerals) or other substances with a nutritional or physiological effect, alone or in combination, marketed in dose form designed to be taken in measured small unit quantities” [19,20]. However, a specific definition of DS intended for pediatric use does not exist. In terms of DS regulations, a child is an ordinary “general consumer”.

Pediatric DS can be identified on the market solely by the use of terms such as “infant”, “junior”, “baby”, “(for) kids”, “(intended for) child/children”, or similar words/phrases in their name, on the label and/or in advertisements. Some are labeled and marketed as “3+”, “from 6 months”, or “for preschoolers” which implies DS suitability for specific age groups, but these age groups are arbitrary and, in most cases, there is no evidence-based rationale behind them. In fact, most of the mentioned terms/phrases are not legally defined. The only age groups clearly defined by both EU and Serbian legislation are infants, as children under 12 months of age, and young children, as children between one and three years. However, these definitions are of higher relevance to infant formulae and foods for infants and young children, which are foods distinctively different from DS, defined by specific regulations in the EU and Serbia [19,21,22,23] which explicitly exclude DS. Nowhere in the legislation are those defined age groups linked to DS, nor are DS classified as (un)suitable for these specific age groups. Therefore, the interpretation of such terms is left to consumers, more specifically, to parents or caregivers.

Pham et al. [8] reported that in Southern Vietnam more than 20% of DS “prescribed” to children of up to 5 years by healthcare professionals were not in compliance with the age limit recommended by the DS manufacturer (were given to children below the recommended age limit). This implies that parents or caregivers are being entrusted with decision-making in which even the more competent, healthcare professionals occasionally make mistakes. Such practice puts the children at risk from misuse of DS and consequent safety issues.

Due to the lack of a single, universally accepted definition, many products are classified differently in different countries outside of the EU (e.g., as foods, DS or drugs, medicinal supplements, medicinal products). Globally, this makes product placement challenging, as manufacturers are forced to adapt to different regulatory requirements of each market. Due to financial and other limitations, these adaptations do not always take place which possibly results in inconsistent compliance to regulations on different markets.

### 3.2. Practical Aspects of Pediatric Dietary Supplements Use

Routine use of DS in healthy children with varied diets (apart from vitamin D in infants) is not recommended [10] but the reported prevalences of DS use are consistently high. Prevalence of DS use among children varies across populations and averages between 20% and 50% or more [11,24,25,26,27,28] but prevalences as high as 79% has been reported in specific age groups [9]. These data call for a coordinated action of all stakeholders—regulators, manufacturers, and healthcare providers—to better regulate this part of the DS market in order to minimize potential risks to children.

The most common reasons for use of DS among children are filling perceived nutrient gaps, improvement of overall health and boosting the child’s immune system [6,10,11,29,30]. The types and reasons differ by age groups, and later in childhood, by gender [31,32,33].

There is currently no universal, evidence-based guidance for rational DS use among children. Consequently, healthcare professionals rely on their individual competencies when advising on the use of DS. Such practice can lead to inconsistent advice being provided by healthcare providers, as their knowledge varies [34], undermining parents’ trust. Adding to the problem, Pham et al. [8] demonstrated that in Southern Vietnam close to 15% of DS “prescribed” to children up to 5 years of age by healthcare professionals exceeded the manufacturer-recommended daily dose of DS ingredients. For this reason, rigorous DS-related research, harmonization of regulation across countries, and implementation of new knowledge into practical guidelines for healthcare providers, coupled with their continuous education, is crucial to ensure the safety of children using DS. An added problem is the fact that DS are readily available to lay consumers via online shops or regular supermarkets—channels that exclude pharmacists or other healthcare professionals with the most insightful knowledge on DS, as middle-men.

Nevertheless, research shows that parents/caregivers still do consult healthcare professionals when considering the use of DS in children [9,10,11,29], but there are several barriers to following professionals’ advice such as inconsistencies, personal beliefs and characteristics, as well as advertisements.

Higher family income is often associated with higher prevalence of DS use among children [10,30,35]. It has been reported that mothers’ own attitudes and practices towards DS heavily influence the use of DS among their children [36]. Multiple studies have shown that DS use is higher in children of parents with higher educational attainment, more specifically, mothers’ education levels [11,25,29,37]. Arguably, it is not the level of education, but the field of education that would help parents or caregivers make better, more informed choices. Nevertheless, recognizing that children’s use of DS, especially in early childhood, is dictated by their parents/caregivers and knowing that mothers are usually family’s primary caregivers, it is clear that women should be the primary target population for educational campaigns concerning the use of DS among younger children.

### 3.3. Undefined or Vague Composition Standards

In general, DS production ought to follow the recommendations of Good Manufacturing Practice (GMP). As this system is allowed to be tailored to each producer’s specific needs, the quality of DS is not universally uniform. Unlike pharmaceuticals, quality standards for DS are not covered by official pharmacopeias in EU or Serbia, which contributes to the variability of their characteristics, including their ingredients.

As there is no specific definition of DS intended for pediatric use, these DS are not subject to any additional or more strict legal requirements concerning their composition compared to DS in general.

EU and Serbian national law detail only the use of vitamins, minerals and, in part, herbal ingredients in DS, indiscriminately of their intended target population. Specifically, vitamins, minerals and vitamins, and mineral substances which can be used in the manufacture of DS are defined [19,20]. Other ingredients in DS, such as probiotics, essential fatty acids, and herbal and other bioactive components, are even more loosely regulated. Their use in DS is allowed, both by EU and Serbian legislation that refers to them as “other substances with a nutritional or physiological effect” [19,20], but does not discuss them further, apart from a comment that specific rules for these will be set at a later time. Use of herbal components in DS is defined in brief in the Serbian DS legislation [20] that specifies that the use of herbal components with strong pharmacological activity or with known harmful effects is not permitted. Herbal components are permitted to contain between 15% and 65% of the lowest recommended daily dose for herbal medicinal products defined in the monographs of the European Medicines Agency (EMA) and other appropriate sources. However, not even these legal requirements are specifically tailored for children. The Committee on Herbal Medicinal Products of EMA provides the overview of recommendations for the uses of herbal medicinal products in the pediatric population [38], but only a few herbal drugs or herbal drug preparations have the well-established or traditional use in children under 12 years. Additionally, age-appropriate dosing is provided only for a limited number of herbs.

The initiative to establish maximum and minimum levels for vitamins or minerals that may be added to food supplements has been started by the European Commission, but its results are still pending [39]. Meanwhile, Serbian national regulation [20] offers a list of maximum amounts of vitamins and minerals which can be contained in a daily dose of DS, but with no mention of other ingredients and other age groups apart from adults. There are currently no EU or national standards or guidelines for pediatric age groups [19,20].

Hence, no globally harmonized rules specify which ingredients and in which amounts are allowed to be present in DS. In addition to the general lack of guidance on doses of DS ingredients, arbitrary age cut-offs and specific needs during different stages of childhood further complicate dosage adjustment for children relative to adults.

In contrast, there are specific requirements [21,22,23] for other dietary products such as infant and follow-on formulae in terms of maximum amounts of proteins, lipids, carbohydrates, vitamins, minerals, and other essential components of these products. For dietary foods intended for infant and young children, the maximum amounts of vitamins, minerals, and trace elements are regulated [21,22]. Although these examples pertain only to specific age groups, they indicate that it is possible to adapt legal systems to protect vulnerable populations such as children. This point is further affirmed by the existence of a separate EU Regulation on medicinal products for pediatric use [40]. So, what are the obstacles for similar systems to be adopted in DS regulation frameworks?

Attempts have been made to classify DS for children by their ingredients [41], but without governmental and other stakeholders’ support, it is highly unlikely that this effort will be realized in the near future.

### 3.4. Potential Safety Risks Related to Ambiguous Composition Standards, Arbitrary Age Cut-Offs, and Lack of Age-Appropriate Reference Values Specific to Dietary Supplements

Use of DS is not without perils. Even DS that completely conforms to the existing regulatory requirements can occasionally present different health risks, from overdosing to possible unknown long-term effects. This is even more true for the pediatric consumers.

The upper safe levels of intake can easily be significantly exceeded for some vitamins/minerals, especially among children, possibly causing harm or unwanted side effects [42]. A study by Woźniak et al. [9] demonstrated the possibility of excessive intake of vitamin A, C, several vitamin Bs, as well as copper while using DS in practice. A study of child-targeted DS from Canada [43] found that median amounts of vitamins A, B6, B12, and C, thiamin, riboflavin, pantothenic acid, and biotin in those DS were higher than Adequate Intake (AI) by a large margin (e.g., up to 5557% for vitamin B12). One US study [44] found that vitamin A and niacin were often present in DS in amounts equal or above the tolerable upper limit for 1 to 3 year-olds. Another market analysis study conducted in the US [45] showed that the majority of DS intended for children up to 4 years old contained vitamins and minerals in amounts exceeding the Recommended Daily Allowance (RDA) or AI (e.g., up to 936% of biotin AI) for the age group in question with the exception of vitamin D and choline. This was against the national recommendation that food, and not DS, should be the only dietary source of vitamins K and B_1_, thiamine, riboflavin, folate, pantothenic acid, and biotin before the age of 4 (and even more vitamins up to 12 months) in order to avoid high levels of intake [46].

A report from Food Supplements Europe [47] applied the risk management approach to establish the maximum levels of vitamins and minerals in DS for children aged 4 to 10. It concluded that there are potential risks associated with excessive intakes of vitamin A, beta-carotene, calcium, copper, iodine, iron, manganese, and zinc with possible effects ranging from mild gastrointestinal discomfort to serious toxicity. Proposed maximum safe levels were established for these, as well as for vitamins B6, C, D, E, nicotinamide, molybdenum, phosphorus, selenium, magnesium, folic acid, and potassium (characterized as low risk of exceeding UL). However, this report deals only with vitamins and minerals, again leaving a lot of other ingredients undiscussed. Additionally, it refers only to children between the ages of 4 and 10 years due to specific concerns linked to puberty, adolescent growth spurt, and concurrent dietary changes that need to be considered in more detail, as stated in the document. In addition, older children often use dietary supplements intended for adults indiscriminately, but the safety of such practice is questionable.

A small study of DS intended for use in children performed in Serbia [48] showed that the amounts of iodine and niacin in daily doses of DS exceed the RDAs for all children except for 14–18 year-old boys and all children younger than 8, respectively.

Contrary to the common belief that herbal products are harmless, due to being natural, there are various potential health risks associated with the use of herbal DS. The composition of herbal drugs or herbal drug preparations could vary greatly or may not be described in enough detail, compromising the safety of herbal DS. In addition, herbal DS for children often lack evidence-based recommendations for age-appropriate use as data on dosing in children is limited.

Ambiguous composition standards, coupled with other regulatory gaps, raise additional safety concerns, especially for children using DS containing herbs or “other substances”. Potential health risks include excessive intake and toxicity, interactions with medications, allergic reactions, or use of substances with no safety profiles determined for pediatric users [8,49]. In this context, probiotic supplements of the most commonly used species seem to present the lowest risks, but theoretical concerns still exist [50].

Special consideration should be given to children with chronic conditions who use DS more often [30,51] and are even more vulnerable to potential side effects and adverse reactions to DS than children with no chronic diseases.

Another safety concern is linked to the excipients used in DS. Several excipients commonly used in DS can potentially cause adverse effects in children. These include saccharose, glucose, aspartame, benzoic acid/salts, sorbitol, xylitol, glycerol, and propylene glycol [50]. Serbian study [48] showed that these excipients were present in 77% of DS intended for use in children at the local market. Unfortunately, this problem is not exclusive to DS, as another study [52] reported similar problems with medicines, namely pediatric nasal medicines.

It is also of interest to note that this study did not consider the risks of DS adulteration and contamination.

### 3.5. Misuse of Pediatric Dietary Supplements Labeling and Marketing Practices

Laws regulating labeling and advertising of foodstuff [53,54] apply to DS with some rules being superseded by the specific requirements for DS, such as declaring net content and including precautionary or warning statements on the label [19,20].

The label and advertisements must never mislead consumers, but when it comes to DS, the situation is far from perfect. For example, one study [55] demonstrated consistent non-compliance of DS radio advertisements in Spain to EU regulations.

Apart from the previously described use of legally unregulated phrases (“for kids”, “junior”, “3+”, etc.), manufacturers frequently omit or provide illegible mandatory information, use non-harmonized reference values (especially for different pediatric age groups), give scientifically unsupported dosing instructions, or use inappropriately reworded authorized or unauthorized health claims on labels [56].

One Spanish study [57] cited “overly optimistic image, lack of scientific basis, and failure to recommend medical supervision” as the main challenges in marketing practices pertaining to DS for children. In addition, labels and advertisements of DS for pediatric use are colorful, often depict cartoon-like characters and are similar to those of sweets and candies, possibly promoting undesirable eating habits in susceptible population groups such as children [58,59]. To digress, considering that many countries are actively limiting advertisement of unhealthy foods [60] in order to curb the unhealthy lifestyles and obesity epidemic among today’s children, formulation of DS into candy-like products (lollipops, gummies) might be a counterproductive move, as it blurs the lines between treats and DS [61].

Adding to the issue of problematic labeling is the failure to label excipients, with some reports stating that excipients were not labeled in 20% of DS intended for children’s use available at the local market [48]. When it comes to medicines, excipients with known actions or effects are required to be labeled. This strategy, if mirrored in DS regulation, could improve the safety of pediatric DS users.

One of the most problematic areas of non-compliance with the regulations are health claims [60,62,63]. The reported prevalence of DS health claims compliance with regulations varies depending on health claim type and the related ingredient, from 10% vitamins- and minerals-related health claims on herbal DS in Serbia [64] to 20% of functional claims on DS in Spain [55].

Use of pompous yet misleading and unauthorized health claims is, therefore, not uncommon. The practice of rephrasing and rewording of authorized health claims to target parents and older children more efficiently adds to the confusion. In general, the rewording can be accepted provided that the original meaning of the claim is not changed but this rule is often misused and the claimed effects are exaggerated, broadened, or adjusted for specific populations such as children.

In addition, health claims used on pediatric supplements’ labels are usually Article 13 claims (general function claims), which refer to the general public, whereas article 14(1)(b) health claims, the ones referring to children’s development and health [65], are seldomly used. Therefore, even the labeling practices that are formally compliant with legislation may mislead and disproportionately influence consumer perceptions, especially for products intended for children, but marketed to their parents/caregivers.

There is limited evidence showing that health-related messages used on the labels and in advertising of DS for children translate into safe and beneficial DS use among children. To add to this issue, a recent study [66] demonstrated that dietary supplements bearing health claims were often perceived as providing a “disease-specific benefit, contrary to their regulatory intent”, further emphasizing the need for clear, parent/caregiver- and/or child-specific practical guidelines on labeling and advertising.

And lastly, media is often cited as one of the factors influencing the decision to use DS or give DS to children (or not) [10,11,58]. The expansion of social media and the practice of marketing DS by influencers with limited or no formal knowledge about DS is currently a hot topic. Due to the potential of their platforms to reach wide audiences, in this case specifically parents and older children, with limited critical capacity, regulations should be actively enforced to restrict such practices as soon as possible. Such regulations are already being implemented in other regions, specifically China [67]. Their experiences and future outcomes could serve as valuable lessons for the European region should similar strategies be adopted in order to mitigate the risks of spreading misinformation via social media.

### 3.6. Strengths and Limitations of the Study

This work offers a comprehensive overview of the main shortcomings of DS regulations concerning the definition, composition, labeling, and advertisement of DS intended for use in a vulnerable population such as children. Inclusion of information from both EU and Serbia highlight specific problems that cross borders, strengthening the need for universal, harmonized legislation that would ensure the same level of safety for all children. This work also points to specific areas where regulatory improvements and updates can be made, and offers practical insights for policymakers.

On the other hand, this study also has some limitations. Its conclusions cannot be globally generalized due to differences in regulatory systems between regions other than Europe which were not considered in this work. This work does not assess the direct effects of regulatory inconsistencies to children’s health, but rather the potential of these inconsistencies to increase safety risks among children.

## 4. Conclusions

This study identified four critical areas of concern within the current regulations for pediatric DS in EU and Serbia: (i) the absence of universal and clear product definition, (ii) the lack of uniform, age-appropriate composition standards, (iii) potential safety risks related to ambiguous composition standards, arbitrary age cut-offs and lack of age-appropriate reference values specific to DS, (iv) misuse of labels and unfair advertising practices, and, an additional point, (v) practical aspects of pediatric DS use and the limited role of healthcare providers in it.

Presented findings suggest that regulating pediatric DS is a complex task due to the diversity of the pediatric population. Regulatory gaps and vagueness, combined with the lack of universal guidelines or recommendations for DS use lead to inconsistencies in advice on DS use provided by healthcare professionals and undermine parents’/caregivers’ trust. On the other hand, use of misleading labels, unauthorized or exaggerated health claims and unfair marketing practices create a high-risk environment for inappropriate DS use among children. Such risks could be alleviated by implementation of stricter overseeing mechanisms. It is clear that one of the first steps in improving the regulation of pediatric DS should be the creation of comprehensive, age-appropriate lists of reference values for vitamins, minerals, as well as other substances commonly present in DS.

Finally, considering the diversity of the pediatric population, from newborns to adolescents, a fit-for-all solution to the issues discussed in this work might not be found in the near future. However, regulatory systems must be ready to resolve all inconsistencies, act swiftly and adjust to new scientific developments and market changes. Active research is essential in order to ensure our primary goal—children’s health and safety.

## Data Availability

Not applicable.

## Supplementary Materials

The following supporting information can be downloaded at: https://www.mdpi.com/article/10.3390/children13010074/s1, Table S1. Overview of the identified themes and subthemes; Table S2. Illustrative examples of thematic coding derived from regulatory documents and selected literature on pediatric dietary supplements.

## Abbreviations

The following abbreviations are used in this manuscript:

DS	Dietary supplements
EU	European Union
RDA	Recommended Daily Allowance
AI	Adequate Intake
UL	Tolerable Upper Intake Level
EMA	European Medicines Agency
