# Supplementary material for: Dietary Supplements’ Regulation: Are We Doing Enough to Protect the Children? A Narrative Review
Source: Children (Basel). 2026 Jan 2;13(1):74. doi: 10.3390/children13010074 (PMC12840536; doi:10.3390/children13010074)
Supplement: Supplementary file 1 [file children-13-00074-s001.zip › children-4066392-supplementary.pdf]

Table S1. Overview of the identified themes and subthemes.

	Theme	Subthemes
1.	Absence of universal and clear pediatric dietary supplement definition	Lack of internationally accepted definition
		Pediatric dietary supplements identified only by marketing terms (e.g., “infant”, “kids”, “3+”)
		Age groups for labeling are arbitrary and not evidence-based
		Misinterpretation of manufacturers’ recommendations for use
2.	Practical aspects of pediatric dietary supplement use	High prevalence of dietary supplement use
		Lack of universal, evidence-based guidance for rational dietary supplement use among children
		Influence of parents/caregivers, socioeconomic and educational factors
		Availability to consumers while bypassing healthcare professionals
3.	Undefined or vague composition standards	No pediatric-specific composition requirements
		Variability in quality and ingredient content
		Lack of harmonized rules globally for dietary supplement ingredients and dosages
		Comparison with infant/young child formula regulations as example
4.	Potential safety risks due to composition standards, age cut-offs, lack of reference values	Risk of exceeding safe vitamin/mineral intake
		Limited evidence for herbal and “other components” safety
		Vulnerable populations
		Risks from excipients
5.	Misuse of pediatric dietary supplement labeling and marketing practices	Non-compliance with labeling/advertising regulations
		Misleading or unsupported health claims
		Influence of media

Table S2. Illustrative examples of thematic coding derived from regulatory documents and selected literature on pediatric dietary supplements.

Source	Evidence/Provision	Code	Category	Final theme
[1]EU Directive 2002/46/EC [2]Serbian Rulebook on Food Supplements, 2022.	Dietary supplements defined as products intended to supplement the diet, without age-specific subcategories	Absence of pediatric-specific legal definition	Regulatory gaps	Absence of universal and clear pediatric dietary supplements definition
[3-5]Multiple studies	Higher family income is often associated with higher prevalence of dietary supplements use among children	Socioeconomic influence	Determinants of pediatric dietary supplement use	Practical aspects of pediatric dietary supplement use
[2]Serbian Rulebook on Food Supplements, 2022.	Only vitamins, minerals, and some herbal components regulated; amino acids, fatty acids, probiotics unregulated	Lack of composition standards	Regulatory gap	Undefined or vague composition standards
[6]Food Supplements Europe, 2021.	Excessive intake of vitamins/minerals can cause toxicity in children aged 4–10	Risk of toxicity	Safety concern	Potential safety risks related to ambiguous composition standards, arbitrary age cut-offs and lack of age-appropriate reference values specific to dietary supplements
[7]Assadourian et al., 2025.	DS bearing health claims often perceived as having "disease-specific benefit"	Misleading claims	Labeling and advertising practices	Misuse of labels and unfair advertising practices

1. European Commission. Directive 2002/46/EC of the European Parliament and of the Council of 10 June 2002 on the approximation of the laws of the Member States relating to food supplements. *Official Journal of the European Communities*, **2002**, *L183* (52), 51–57.
2. Rulebook on Food Supplements. *Official Gazette of the Republic of Serbia*, **2022**, *45*.
3. Barretto, J. R.; Gouveia, M. A. da C.; Alves, C. Use of Dietary Supplements by Children and Adolescents. *Jornal de Pediatria*. Elsevier Editora Ltda March 1, **2024**, pp S31–S39. https://doi.org/10.1016/j.jped.2023.09.008
4. Piórecka, B.; Holko, P.; Olesiak, W.; Sekulak, K.; Cichocka-Mroczek, E.; Stąpor, D.; Kosowska, K.; Kawalec, P. Predictors of Dietary Supplement Use Among Children Attending Care and Educational Institutions in Krakow, Poland. *Nutrients*, **2024**, *16* (21), 3662. https://doi.org/10.3390/nu16213662.
5. Mishra, S.; Stierman, B.; Gahche, J. J.; Potischman, N. Dietary Supplement Use among Adults: United States, 2017-2018. *NCHS Data Brief*, **2021**, No. 399, 1–8. https://doi.org/10.15620/cdc:101131
6. Food Supplements Europe. *Risk Management Approaches to the Establishment of Maximum Levels of Vitamins and Minerals in Food Supplements for Adults and for Children Aged 4-10 Years*; 2021.
7. Assadourian, J.N.; Peterson, E. D.; Navar, A. M. Label Statements and Perceived Health Benefits of Dietary Supplements. *JAMA Netw Open,* **2025**, 8 (9), e2533118. doi: 10.1001/jamanetworkopen.2025.33118.

All references cited in this Supplementary Material are also cited in the main manuscript. The references are renumbered here starting from one, for clarity and ease of reading, and therefore do not correspond numerically to the reference numbering in the main text.
